# Benchmarking GPT-5 in radiation oncology: measurable gains, but persistent need for expert oversight

**DOI:** 10.3389/fonc.2025.1695468

**Published:** 2025-12-11

**Authors:** Ugur Dinç, Jibak Sarkar, Philipp Schubert, Sabine Semrau, Thomas Weissmann, Andre Karius, Johann Brand, Bernd-Niklas Axer, Ahmed Gomaa, Pluvio Stephan, Ishita Sheth, Sogand Beirami, Annette Schwarz, Udo Gaipl, Benjamin Frey, Christoph Bert, Stefanie Corradini, Rainer Fietkau, Florian Putz

**Affiliations:** 1 Department of Radiation Oncology, University Hospital Erlangen, Friedrich-Alexander-Universität Erlangen-Nürnberg, Erlangen, Germany; 2 Comprehensive Cancer Center Erlangen-Europäische Metropolregion Nürnberg (European Metropolitan Region Nuremberg) (CCC ER-EMN), Erlangen, Germany; 3 Bavarian Cancer Research Center (BZKF), Munich, Germany; 4 Department of Radiation Oncology, University Hospital, Ludwig Maximilian University of Munich, Munich, Germany

**Keywords:** GPt-5, artificial intelligence, radiation oncology, large language models, treatment recommendation, oncologic decision support, hallucination, real-world evaluation

## Abstract

**Introduction:**

Large language models (LLM) have shown great potential in clinical decision support and medical education. GPT-5 is a novel LLM system that has been specifically marketed towards oncology use. This study comprehensively benchmarks GPT-5 for the field of radiation oncology.

**Methods:**

Performance was assessed using two complementary benchmarks: (i) the American College of Radiology Radiation Oncology In-Training Examination (TXIT, 2021), comprising 300 multiple-choice items, and (ii) a curated set of 60 authentic radiation oncologic vignettes representing diverse disease sites and treatment indications. For the vignette evaluation, GPT-5 was instructed to generate structured therapeutic plans and concise two-line summaries. Four board-certified radiation oncologists independently rated outputs for correctness, comprehensiveness, and hallucinations. Inter-rater reliability was quantified using Fleiss’ *κ*. GPT-5–14 results were compared to published GPT-3.5 and GPT-4 baselines.

**Results:**

On the TXIT benchmark, GPT-5 achieved a mean accuracy of 92.8%, outperforming GPT-4 (78.8%) and GPT-3.5 (62.1%). Domain-specific gains were most pronounced in dose specification and diagnosis. In the vignette evaluation, GPT-5’s treatment recommendations were rated highly for correctness (mean 3.24/4, 95% CI: 3.11–3.38) and comprehensiveness (3.59/4, 95% CI: 3.49–3.69). Hallucinations were rare, flagged in 10.0% of all individual reviewer assessments (24 of 240), and no patient case reached majority consensus for their presence. Inter-rater agreement was low (Fleiss’ *κ* 0.083 for correctness), reflecting inherent variability in clinical judgment. Errors clustered in complex scenarios requiring precise trial knowledge or detailed clinical adaptation.

**Discussion:**

GPT-5 clearly outperformed prior model variants on the radiation oncology multiple-choice benchmark. Although GPT-5 exhibited favorable performance in generating real-world radiation oncology treatment recommendations, correctness ratings indicate room for further improvement. While hallucinations were infrequent, the presence of substantive errors underscores that GPT-5-generated recommendations require rigorous expert oversight before clinical implementation. In addition, considerable inter-rater variability highlights the challenge of achieving consistent expert evaluation.

## 1 Introduction

Large language models (LLMs) have advanced rapidly in recent years, driven by scaling of parameters (1), reinforcement-learning–based alignment (2, 3), and the development of modular architectures such as Mixture-of-Experts (MoE) (4, 5). These innovations have enabled broad use of LLMs across scientific and clinical domains (6–8). In biomedicine, domain-specific pretraining and clinical fine-tuning have enhanced representation of medical terminology and workflows (9–11), while general-purpose models have achieved exam-level performance in several evaluations (12–14). Nonetheless, accuracy remains heterogeneous across specialties and problem types (15–18). Current consensus emphasizes transparent communication of model limitations and the need for sustained human oversight (17, 18).

Within radiation oncology, deep learning methods are established for tasks such as segmentation, image enhancement, dose estimation, and outcome prediction (19–25). LLMs extend this toolkit with text-centric applications such as guideline summarization, structured rationale generation, question answering, and automated documentation (26–28). Evaluation of such models now include physics-focused question sets (29, 30) as well as surgical and board-style assessments (31, 32). Radiation oncology-specific studies underscore both the promise of LLMs and the persistence of domain-specific limitations (e.g., dose prescription, differentiation between percutaneous and interstitial techniques) as well as challenges in keeping pace with evolving trial evidence (16, 17).

A key area of ongoing research is the use of large language models as clinical decision support (CDS) systems, with prominent initiatives including Med-PaLM, Med-PaLM 2, and Google AMIE (13, 33, 34). LLM agents like AMIE illustrate how LLM-based assistants may retrieve, synthesize, and contextualize medical evidence for patient-specific recommendations under expert supervision (33). Early evaluations report promising accuracy in case-based reasoning and treatment planning, while underscoring the necessity of explicit uncertainty handling and clinician oversight (16, 18, 35).

GPT-5, the latest generation of OpenAI’s foundation models, represents a fundamental shift compared to GPT-3.5 and GPT-4 by explicitly incorporating reasoning-focused reinforcement learning reward models (36). In combination with a larger MoE backbone and improved calibration of probabilistic outputs, GPT-5 achieves stronger logical consistency, longer-context reasoning, and higher factual accuracy. Importantly, GPT-5 is a major OpenAI model explicitly positioned as a reasoning model, designed to generate structured, interpretable rationales in addition to predictions. These advances have translated into improved performance across biomedical benchmarks, USMLE-style exams, radiology case reasoning, and oncology-specific tasks, while also reducing hallucination rates (17, 18, 37, 38). Despite these improvements, supervised use remains essential, particularly in high-stakes oncology settings.

Building on this progress, the present work provides the first comprehensive evaluation of GPT-5 in radiation oncology. We investigate two complementary settings: (i) a benchmark against the American College of Radiology Radiation Oncology In-Training Examination (ACR TXIT) subset using an automated Responses API pipeline directly comparable to GPT-3.5/4 results, and (ii) a novel real-world scenario dataset comprising 60 complex clinical cases without a single established standard of care (39).

By jointly analyzing standardized benchmarking and novel scenario-based evaluation, we assess GPT-5’s accuracy, comprehensiveness and hallucination frequency. This dual design allows rigorous quantification of performance while also examining GPT-5’s practical usability and failure modes in clinically ambiguous situations. Given the explicit positioning of GPT-5 as a reasoning model for scientific and medical tasks, our study provides a timely and domain-specific benchmark of its potential and limitations in radiation oncology.

## 2 Materials and methods

This study comprised two complementary evaluations of GPT-5 in radiation oncology: (i) performance on a standardized multiple-choice knowledge benchmark, and (ii) structured decision-support recommendations on real-world oncologic case vignettes. All analyses were performed on de-identified data, using isolated sessions without cross-case information transfer. No protected health information was processed.

### 2.1 GPT-5 model and prompting framework

The large language model (LLM) under test corresponds to the GPT-5 family as characterized in the publicly released system card (37). The specific model identifier is gpt-5-2025-08-07. GPT-5 is a transformer-based model trained on large text corpora with mixture-of-experts routing and reinforcement learning from human and artifical intelligence (AI) feedback. For reproducibility, standardized instructions were used for all experiments, and prompts/outputs were logged. Each API call was executed in a fresh session to avoid context leakage between cases. Automation was implemented in Python.

### 2.2 ACR radiation oncology in-training examination benchmark

Knowledge-based performance was assessed using the 2021 American College of Radiology (ACR) Radiation Oncology In-Training Examination (TXIT) (39, 40), which comprises 300 multiple-choice questions spanning statistics, physics, biology, and clinical radiation oncology across disease sites. Fourteen questions included medical images, of which seven required visual interpretation (Q17, Q86, Q112, Q116, Q125, Q143, Q164). Since only GPT-5 is capable of processing image-based items, these questions were included exclusively in its evaluation. For GPT-3.5 and GPT-4, the image-based items were removed prior to testing, and the total number of eligible questions was adjusted accordingly, resulting in 293 scorable items, consistent with prior work (39). The 2021 TXIT version was retained to ensure comparability and consistency with previous benchmark studies of GPT-3.5 and GPT-4.

Questions were presented as stem plus options without auxiliary text. Prompts instructed the model to select exactly one option and return the format Final answer: X with *X* ∈ {*A,B,C,D*}. No external tools (especially web search) or interactive feedback were permitted. Scoring followed established methodology: 1.0 for a correct choice and 0.0 otherwise.

For content analysis, items were mapped to ACR knowledge domains and to a clinical care-path framework (diagnosis, treatment decision, treatment planning, prognosis, toxicity, brachytherapy, and dosimetry) (40). Items explicitly referencing major clinical trials or guidelines (e.g., Stockholm III, CRITICS, PORTEC-3, ORIOLE, AJCC 8th edition) were flagged for subgroup reporting (41–43). Results were compared directly to published GPT-3.5 and GPT-4 benchmarks.

To assess clinical usability, we curated a set of 60 anonymized oncologic case vignettes representing a broad spectrum of disease sites and treatment indications, including definitive, adjuvant, salvage, palliative, and reirradiation scenarios. Source cases were sampled from patients treated in 2025 and subsequently stripped of all identifiers. Patient name, birth date, and ID were removed automatically, while age and sex were retained. Clinical information such as diagnosis, stage, grading, comorbidities, and oncologic history was condensed by two physicians into vignettes, ensuring privacy while preserving clinical representativeness.

The final case set was designed to be balanced across tumor sites and treatment contexts (**Table 1**). Specifically, it included 10 brain tumor cases (glioblastoma, lower-grade glioma, meningioma, vestibular schwannoma, paraganglioma), 10 breast cancer cases (stratified by nodal status, recurrence, and DCIS), 10 lung cancer cases (NSCLC stage III, SBRT, reirradiation, SCLC), 10 rectal/anal cancer cases (neoadjuvant rectal, definitive anal, local recurrence), 10 prostate cancer cases (risk-adapted definitive therapy, biochemical recurrence, local recurrence post-prostatectomy), and 10 metastatic cases (brain, bone, and SBRT). This stratification allowed for evaluation of GPT-5 across both common and complex clinical scenarios, covering a representative cross-section of real-world radiation oncology practice.

**Table 1:** Sampling frame for the 60 clinical cases set.

Category	Subcategory	Number of cases	% of total
Brain Tumor	Glioblastoma (grade 4)	2	3.3%
	Glioma (grade 2/3)	2	3.3%
	Meningioma	2	3.3%
	Vestibular schwannoma	2	3.3%
	Paraganglioma	2	3.3%
Breast Cancer	Adjuvant—nodal positive	2	3.3%
	Adjuvant—nodal negative	2	3.3%
	Adjuvant—low risk	2	3.3%
	Loco-regional recurrence	2	3.3%
	DCIS	2	3.3%
Lung Cancer	NSCLC—definitive (stage III)	3	5.0%
	NSCLC—SBRT	3	5.0%
	NSCLC—reirradiation	2	3.3%
	SCLC	2	3.3%
Rectal/Anal Cancer	Neoadjuvant—rectal cancer	4	6.7%
	Definitive—anal cancer	3	5.0%
	Local recurrence	3	5.0%
Prostate Cancer	Definitive—low risk	2	3.3%
	Definitive—intermediate risk	2	3.3%
	Definitive—high risk	2	3.3%
	Biochemical recurrence after RPE	2	3.3%
	Local recurrence after RPE + EBRT	2	3.3%
Metastases	Brain metastases	4	6.7%
	Palliative—bone metastases	3	5.0%
	SBRT	3	5.0%

DCIS, ductal carcinoma *in situ*; NSCLC, non–small cell lung cancer; SCLC, small-cell lung cancer; SBRT, stereotactic body radiotherapy; RPE, radical prostatectomy; EBRT, external beam radiotherapy.

Category totals were balanced to support stratified analyses by site and radiooncologic treatment indication.

### 2.3 Real-world oncologic decision-support benchmark

Each vignette was paired with a standardized instruction asking GPT-5 to propose a single most appropriate therapeutic plan and briefly justify the recommendation. Required elements included: disease stage, treatment intent, prior therapy, modality/technique, dose/fractionation, target volumes and OAR constraints, expected toxicities, and follow-up considerations. In addition, GPT-5 was instructed to generate a concise two-line summary of the proposed management, which was ultimately used for evaluation.

Vignettes were curated for diversity with the primary criterion of ensuring sufficient representation across the categories shown in **Table 1**. After project initiation, we included the first occurrences of eligible cases within each category until target counts were reached. Cases were logged by category at intake, and their presentation order was randomized prior to model evaluation and expert review to minimize order effects, carryover/anchoring bias, and any unintended adaptation by the model or raters.

Outputs were evaluated by four senior radiation oncologists from a tertiary university hospital. Correctness and comprehensiveness were rated on 4-point Likert scales (4 = fully correct/comprehensive, 1 = not clinically justifiable). Hallucinations were flagged per case by each reviewer (binary). For analysis, we computed the *hallucination score*, defined as the mean fraction of reviewers flagging a hallucination across cases (range 0–1). Consensus thresholds were summarized at levels of any (≥1/4 raters), majority (≥2/4), strong (≥3/4), or unanimous (4/4). Inter-rater agreement was estimated using Fleiss’ *κ* for correctness, comprehensiveness, and hallucination scores (**Table 2**).

**Table 2:** Clinical cases: case-level outcomes across raters.

Metric	Estimate	95% CI	Notes
Correctness (mean/4)	3.24	3.11–3.38	mean across cases
Comprehensiveness (mean/4)	3.59	3.49–3.69	mean across cases
Hallucination (mean % per case)	10.0%	6.8–13.2%	proportion of raters per case
Any hallucination (per case)	40.0%	28.6–52.6%	24/60 cases
Majority/Strong/Unanimous	0%/0%/0%	–	no case ≥2/4, ≥3/4, or 4/4
Inter-rater reliability (Fleiss’)	0.083/0.016/0.111	–	correctness/compreh./hallucination

Exploratory subgroup analyses stratified cases by disease site, prior radiotherapy, and treatment intent. Endpoints included correctness, comprehensiveness and hallucination rate.

Below we provide two illustrative vignettes with GPT-5 therapy suggestions; the complete list of benchmark cases is available in the **Supplementary Material**. Note that dates have been removed from the published vignettes.

Example 1 – Case 2 (83-year-old female)

*Diagnosis:* Rectal carcinoma (5–11cm from anal verge, 1cm above linea dentata), cT4b cN2b cM0, CRM+, EMVI+, G2, mismatch repair–proficient (pMMR).

*Anamnesis and work-up:* ##.##.####: Rectoscopy with biopsy → adenocarcinoma, G2, pMMR (MLH1, MSH2, MSH6, PMS2 intact). ##.##.####: CT chest/abdomen → cT3 cN+ cM0, no distant metastases. ##.##.####: Pelvic MRI → cT4b N2b, CRM+, EMVI+, suspected vaginal infiltration. ##.##.####: Multidisciplinary tumor board recommended total neoadjuvant therapy. ##.##.####: Transvaginal ultrasound → no sonographic vaginal invasion, fat plane preserved.

*GPT-5 suggestion (TNT, long course):* Radiochemotherapy with 50.4Gy/28 fractions (IMRT) plus capecitabine 825mg/m^2^ BID (Mon–Fri), consolidation chemotherapy with mFOLFOX6 q14d ×4 cycles, followed by total mesorectal excision (TME) 8–12 weeks post-RCT.

Example 2 – Case 52 (male, 52 years).

*Diagnosis:* Prostate carcinoma cT1c cN0 cM0, Gleason 4 + 3 = 7b, iPSA 10.8ng/ml, estimated lymph node risk (YALE) ∼7%.

*Anamnesis and work-up:* ##.##.####: Prostate biopsies → poorly differentiated adenocarcinoma, max. Gleason 7b (4 + 3). Since ##.####: LHRH 3-monthly depot. ##.##.####: Tumor board (urologic cancers) → recommendation for local curative therapy, patient in good general condition. ##.##.####: Prostate MRI (pelvis) → no suspicious lymph nodes, indeterminate bone lesion (likely atypical hemangioma), MRI performed for radiotherapy planning. ##.##.####: Uncomplicated implantation of 3 fiducial gold markers.

*GPT-5 suggestion (curative RT + short-term ADT):* Definitive radiotherapy to prostate ± proximal seminal vesicles, 60Gy in 20 fractions (IG-IMRT/VMAT with daily IGRT); no elective pelvic lymph node irradiation; short-term ADT for 4–6 months (if already >6 months, terminate now).

### 2.4 Statistical analysis

For the TXIT benchmark, we report overall accuracy and descriptive breakdowns by domain, care-path category, and trial/guideline-anchored items. For the clinical decision-support benchmark, primary endpoints were expert-rated correctness, comprehensiveness, hallucinations, and concordance with delivered care. Exploratory subgroup analyses were prespecified; no multiplicity adjustment was applied. All automation, randomization seeds, prompts, and raw outputs are provided as **Supplementary Material**. Use of de-identified, retrospective vignettes complied with institutional policies for research on non-human-subjects data.

## 3 Results

### 3.1 Overall TXIT performance

Across the 293 scorable items of the ACR TXIT (2021), previously reported baselines reproduced web–interface performance of 63.1% for GPT-3.5 and 74.1% for GPT-4. Using the application programming interface (API) with a fixed prompt over five repeated runs, GPT-3.5 achieved 62.1% ± 1.1% and GPT-4 78.8% ± 0.9%, consistent with earlier reports (39).

For GPT-5, we conducted five independent runs that included both text-only and image-based questions. Overall accuracy ranged from 92.3% to 93.0%, with a mean of 92.8%. Performance on the subset of image-based items was lower, with only 2 of 7 questions answered correctly. Given that the item pool, scoring criteria, and adjudication procedures were identical to those used for prior models, the observed improvement in accuracy reflects genuine advances in model capability rather than differences in test format or evaluation methodology.

### 3.2 Domain-wise analysis

**Figure 1:**
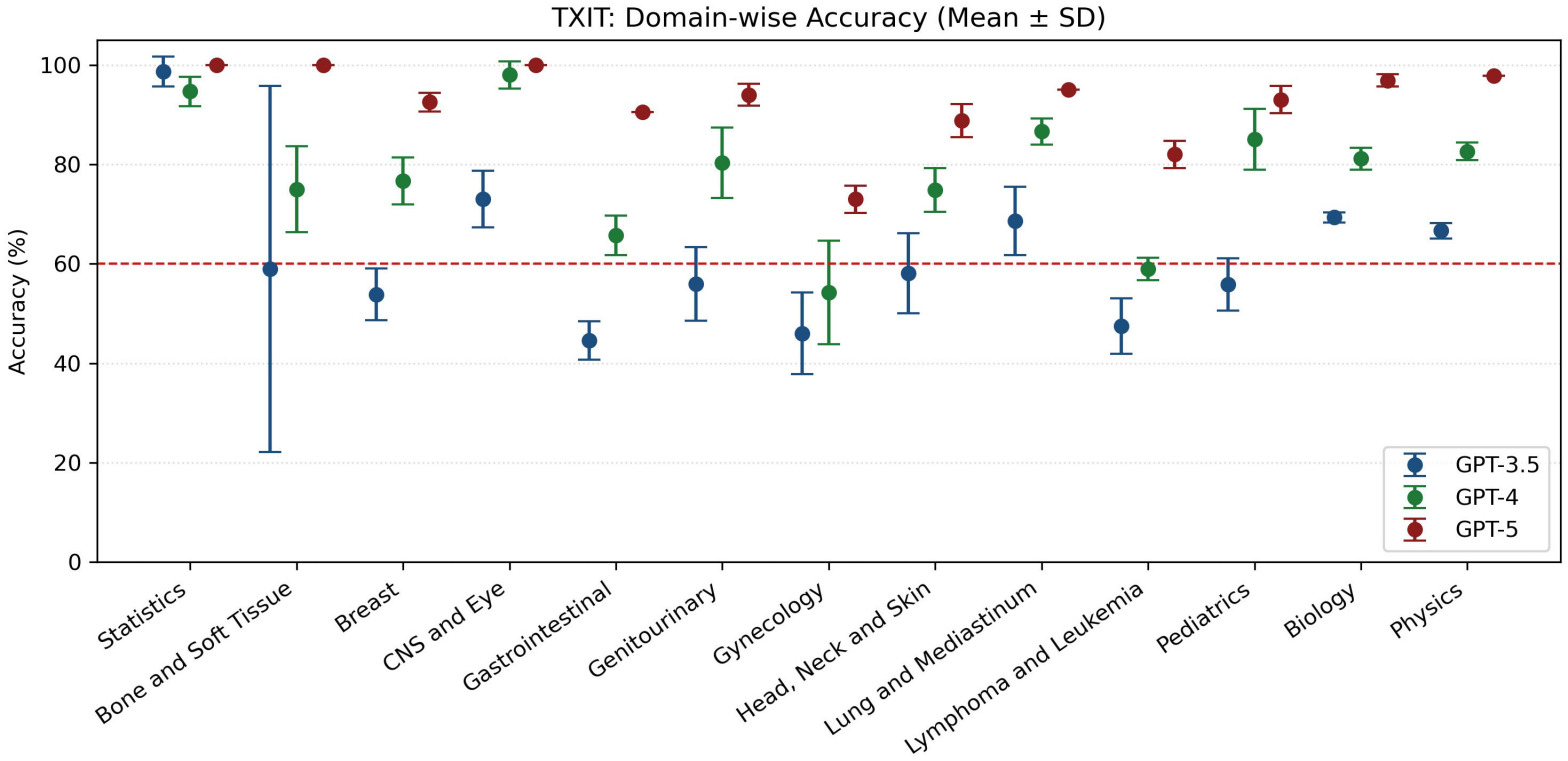
Comprehensiveness ratings (1–4 scale) by tumor group. All groups except for rectal/anal achieved high median scores (≥3.5). Breast, prostate, and brain tumor cases were most consistently rated as highly comprehensive, while rectal/anal and lung cancers showed broader variability.

Stratified by ACR knowledge domains, GPT-5 preserved historical strengths, reaching at least 95% accuracy in Statistics, CNS/Eye, Biology, and Physics. Performance remained lower for Gynecology (75.0%), and moderately reduced for Gastrointestinal and Genitourinary topics (both around 90%). Compared with GPT-4, the largest absolute gains were observed in Dose (from 59.4% to 87.5%) and Diagnosis (from 76.5% to 91.2%) (**Figure 1**).

### 3.3 Clinical care-path analysis

**Figure 2:**
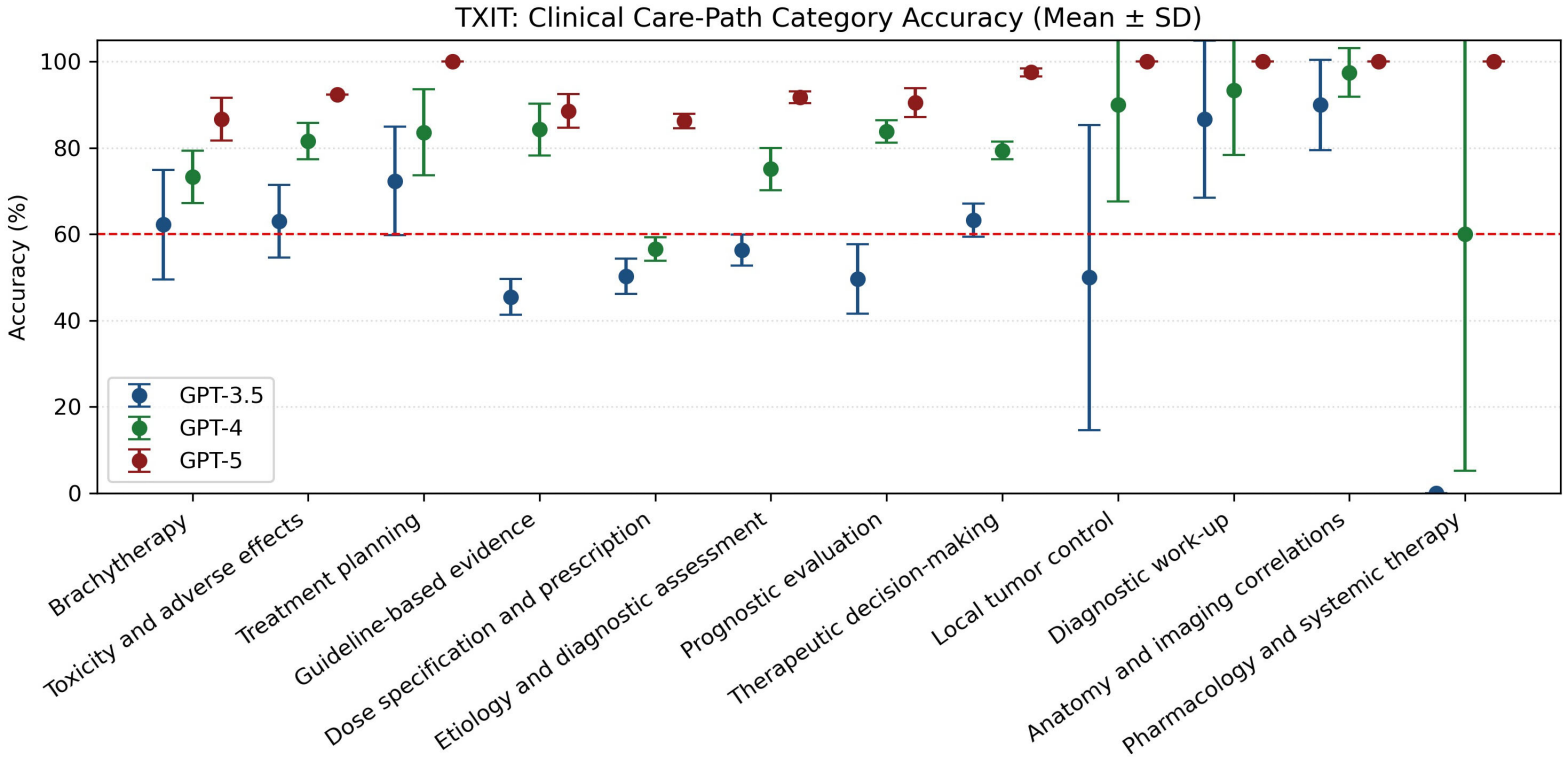
Correctness ratings (1–4 scale) by tumor group. Prostate and brain tumor cases scored highest for correctness (median ≥3.5). Lung and rectal/anal cancer cases showed lower and more variable correctness, whereas breast cancer and metastasis cases performed intermediately.

When mapped to clinical care-path categories, GPT-5 demonstrated consistently high accuracy (**Figure 2**):

- 100% in Treatment Planning (11/11), Local Control (2/2), Diagnosis Methodology (3/3), Anatomy (8/8), and Pharmacology (1/1).
- 95.9% (47/49) in Treatment Decision and 95.2% (20/21) in Prognosis Assessment.
- 92.3% in Toxicity, 92.9% in Trial/Study/Guideline, and 91.2% in Diagnosis.
- 88.9% in Brachytherapy and 87.5% in Dose.

On items explicitly anchored to named trials or staging systems, GPT-5 achieved 92.9% (13/14), outperforming GPT-4 (85.7%) and GPT-3.5 (50.0%).

### 3.4 Evaluation on clinical case vignettes

**Figure 3:**
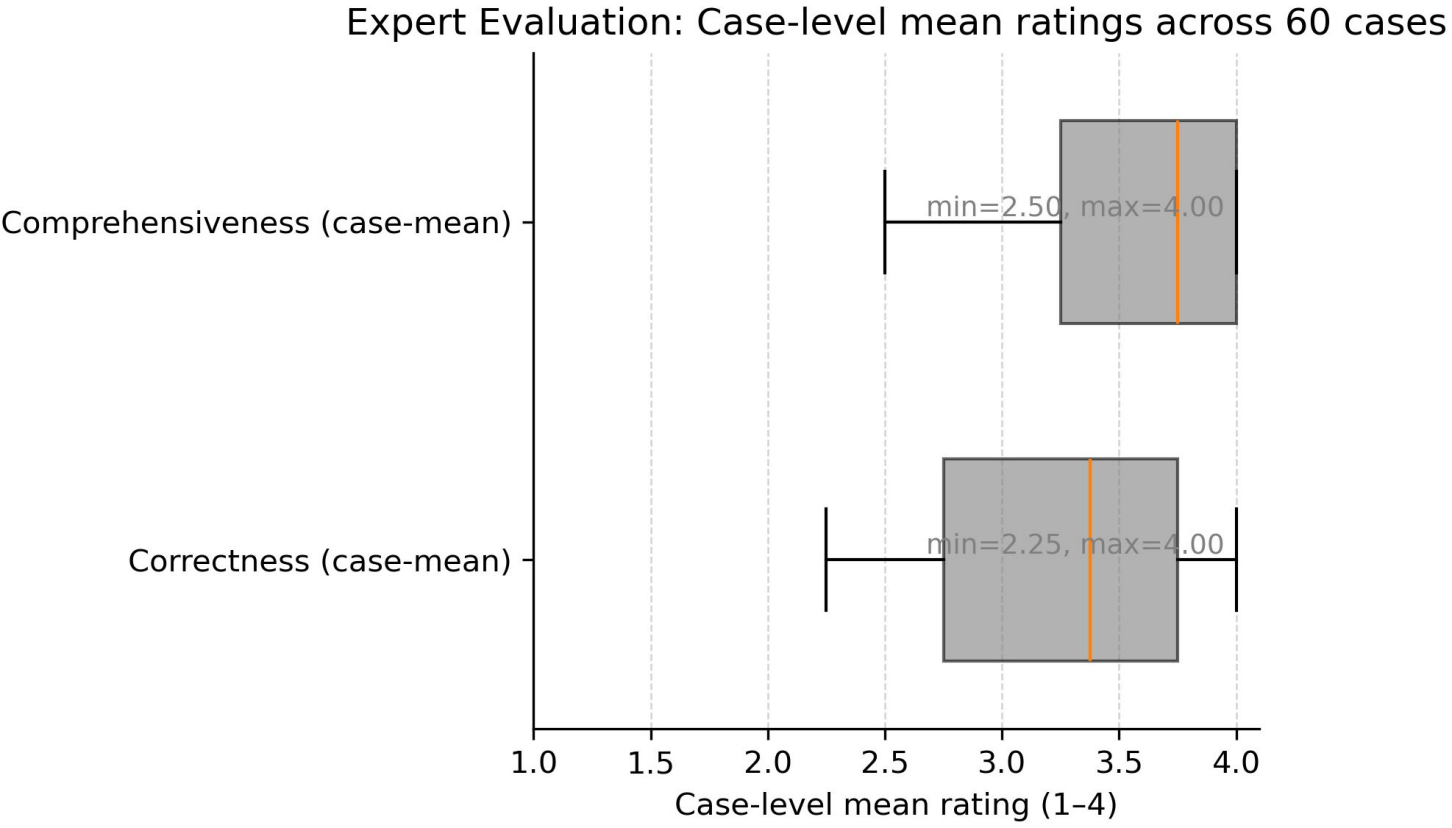
Domain-wise accuracy across models. Symbols show mean accuracy and error bars indicate the SD across five runs for GPT-3.5, GPT-4, and GPT-5.

In the evaluation of 60 real-world radiation oncology vignettes, GPT-5’s treatment recommendations were rated as follows (**Figure 3**):

- Correctness: mean 3.24/4 (95% CI: 3.11–3.38).
- Comprehensiveness: mean 3.59/4 (95% CI: 3.49–3.69).

**Figure 4:**
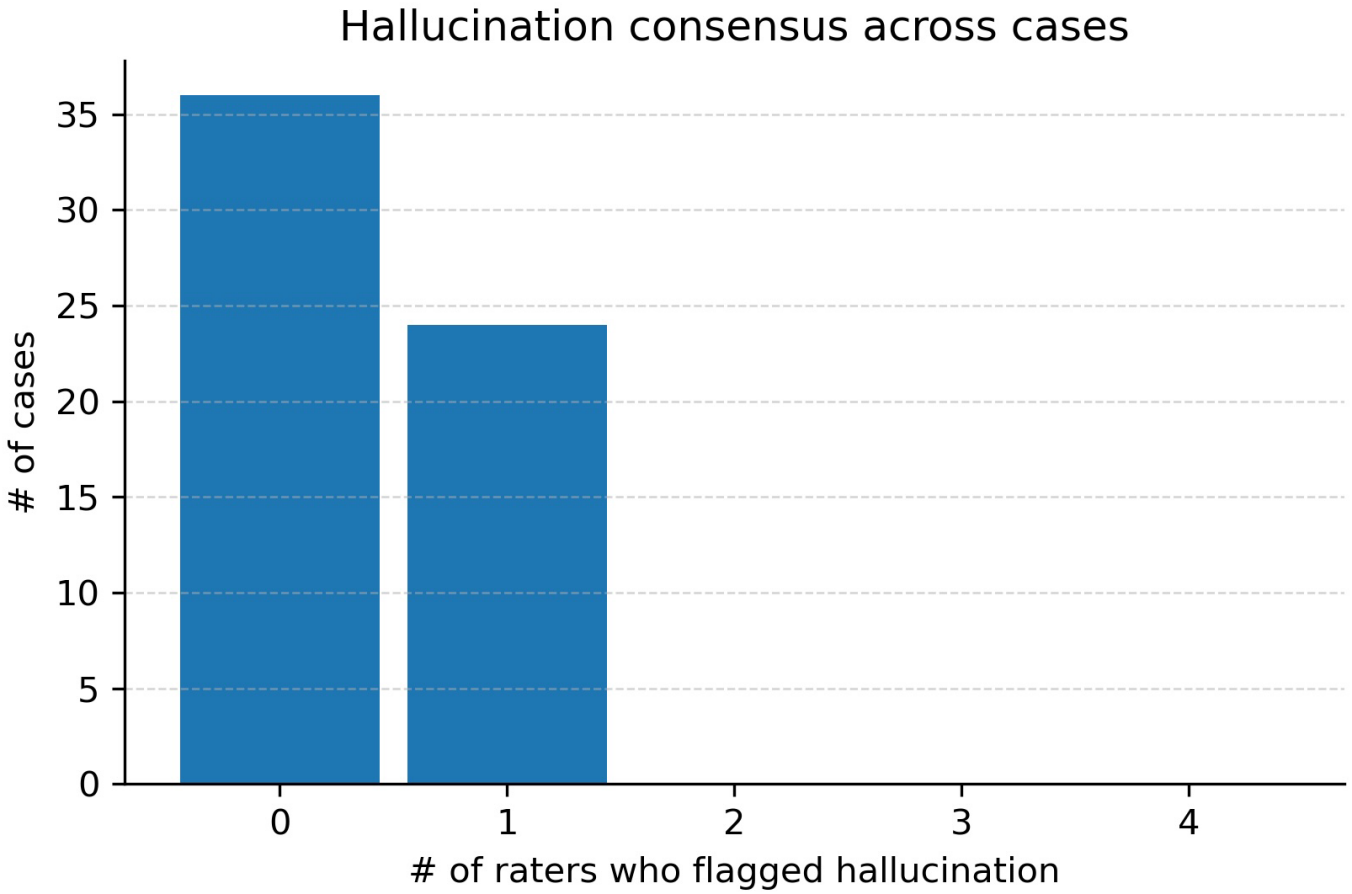
TXIT accuracy by model. Symbols show mean accuracy and error bars indicate the standard deviation (SD) across five runs for GPT-3.5, GPT-4, and GPT-5.

Hallucinations were infrequent. In total, 24 of 240 individual ratings (60 cases × 4 raters) were classified as hallucinations, corresponding to an overall hallucination rate of 10%. Thus, the vast majority of ratings (90%) did not identify hallucinations, indicating that such occurrences were infrequent across cases. No case was flagged by the majority of experts (at least two out of four raters). Distribution was 36/60 (60%) with zero hallucination flags and 24/60 (40%) with exactly one (**Figure 4**).

Inter-rater reliability was low, with Fleiss’ *κ* = 0.083 for correctness, *κ* =−0.016 for comprehensiveness, and *κ* =−0.111 for hallucinations, indicating variability in individual reviewer judgments.

**Figure 5:**
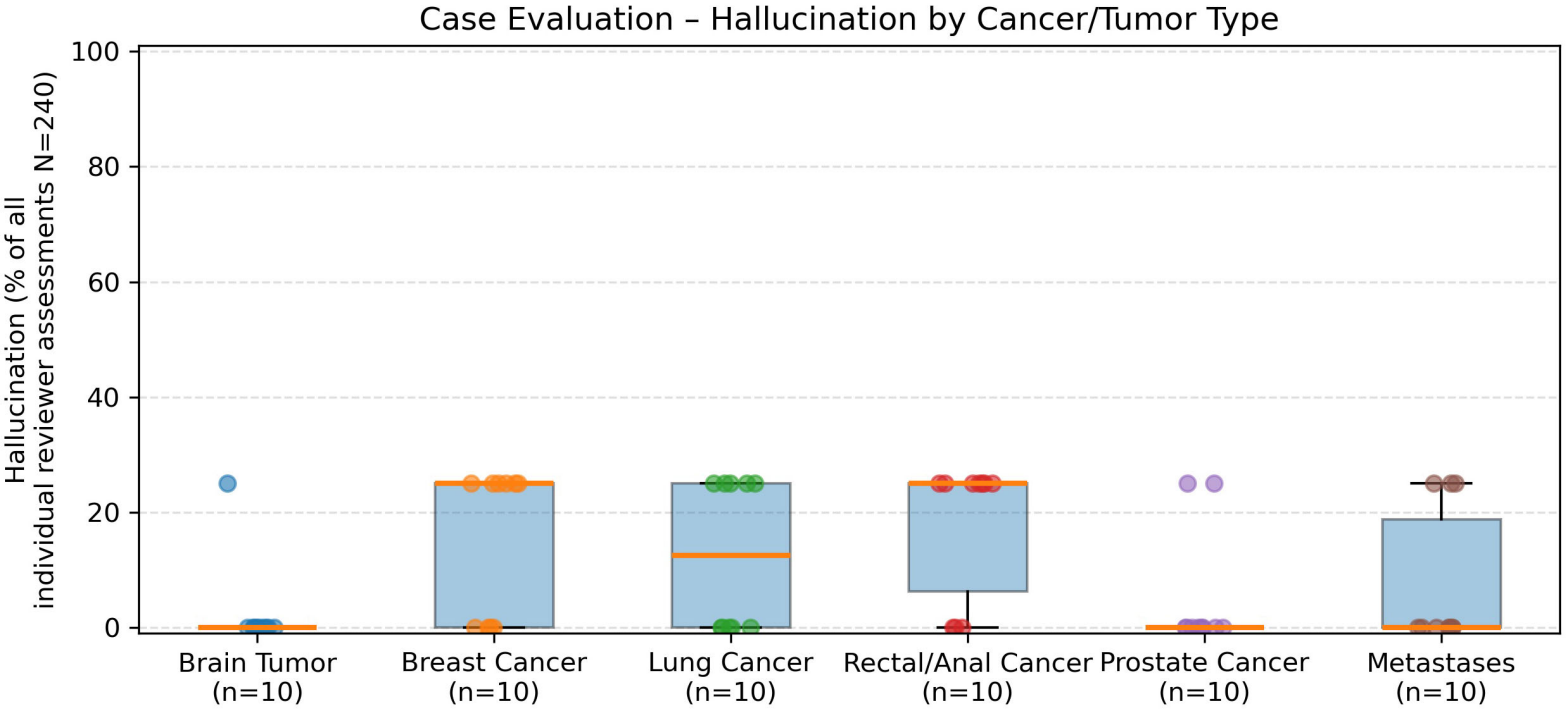
Distribution of case-level mean expert ratings for correctness and comprehensiveness across 60 cases. Each box represents the interquartile range (IQR) with whiskers and outliers, summarizing the spread of ratings. Case-level mean correctness ranged from 2.25 to 4.00, while case-level mean comprehensiveness ranged from 2.50 to 4.00.

**Figure 6:**
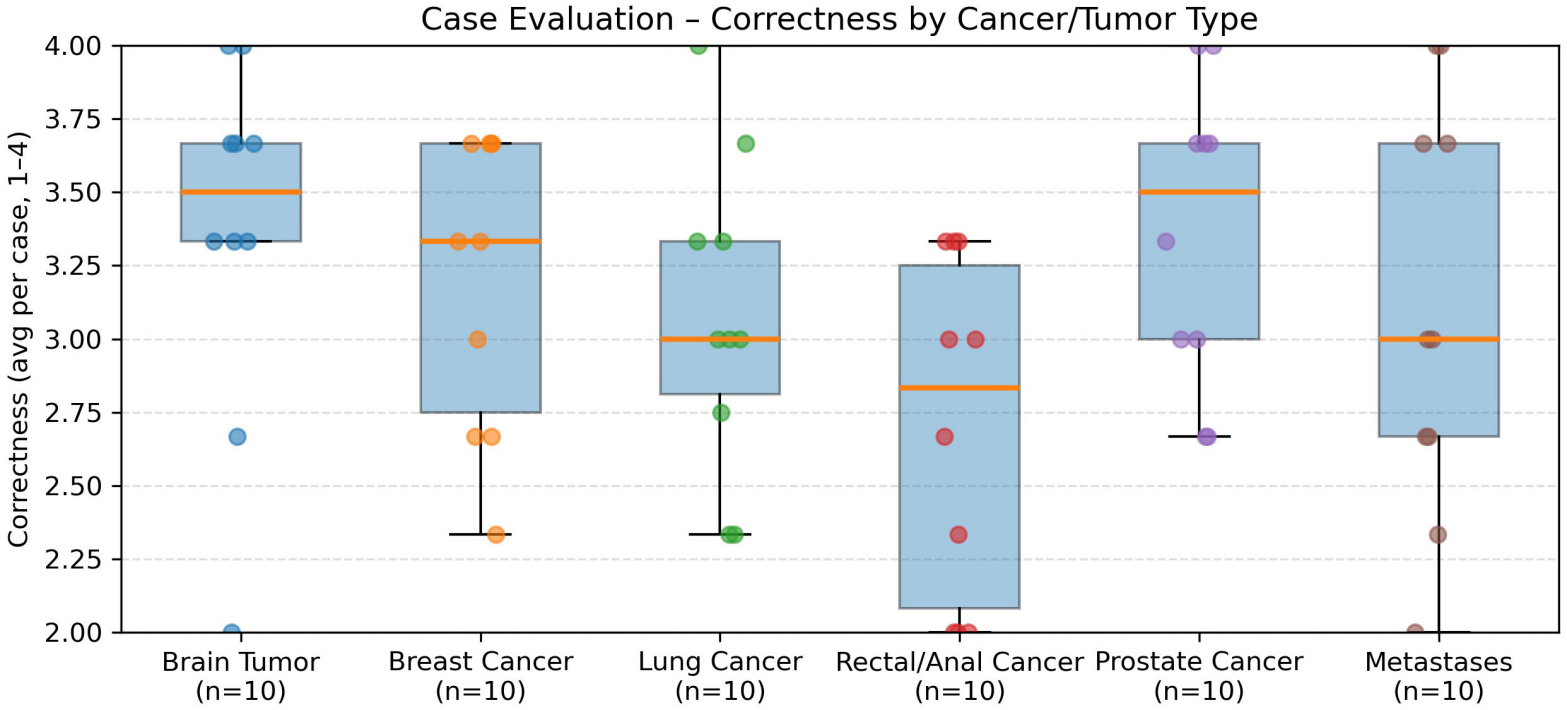
Hallucination consensus across cases. Bars show the number of cases with 0, 1, 2, 3, or 4 raters flagging hallucination. In this cohort, 36/60 cases had 0 flags and 24/60 had exactly 1 flag; no case reached majority (≥2/4), strong (≥3/4), or unanimous (4/4) consensus.

**Figure 7:**
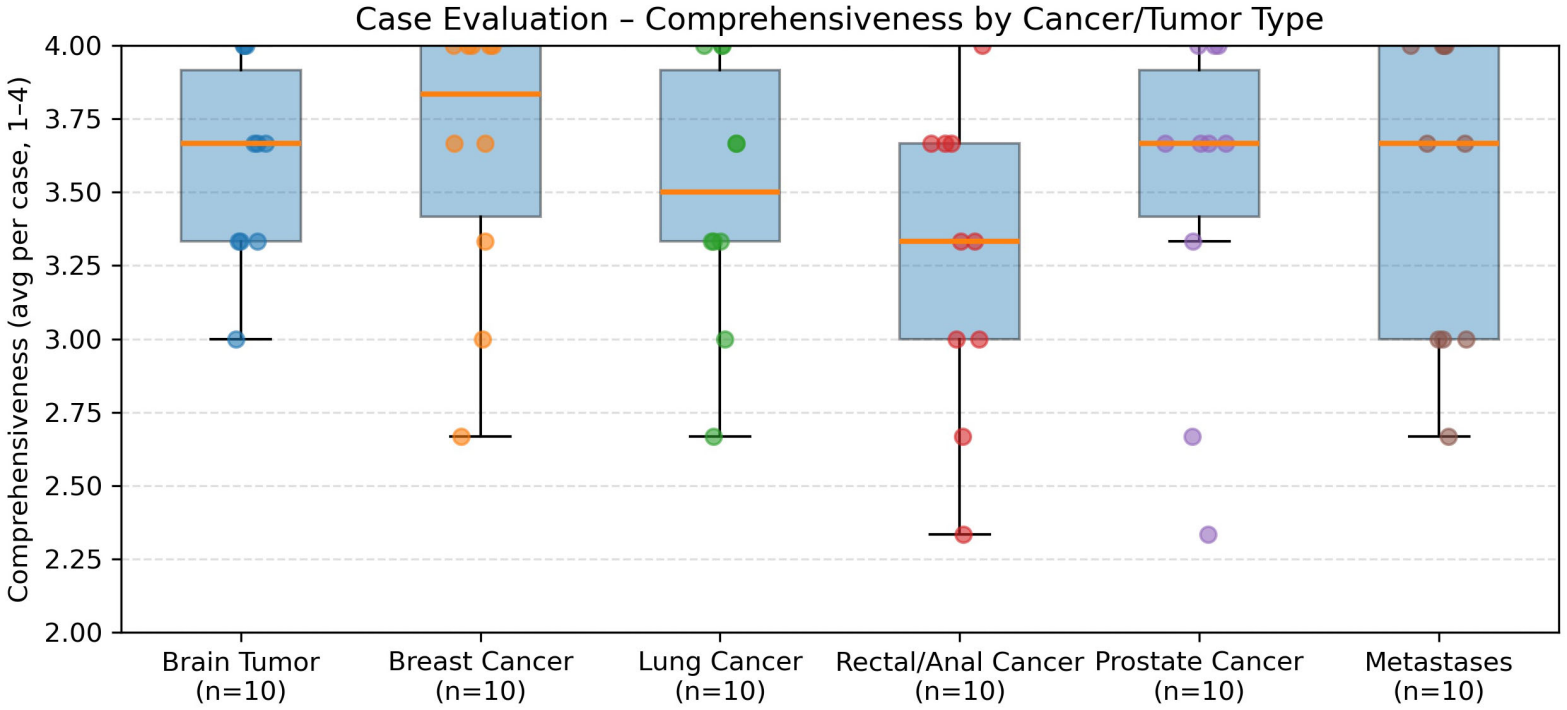
Hallucination rates by tumor group (10 cases each). Hallucinations were rare overall. Prostate and brain tumor cases showed almost no hallucinations, while breast, rectal/anal, lung, and metastasis cases exhibited higher variability, with some reaching up to 25%.

When stratified by the six major tumor groups, distinct patterns emerged (**Figures 5**–**7**). Hallucinations were rare overall, with prostate and brain tumor cases showing almost none, whereas breast, rectal/anal, lung, and metastasis cases exhibited higher variability (**Figure 5**). Comprehensiveness was generally high across all groups (median ≥3.5/4), with breast, prostate, and brain tumors rated most consistently complete, and rectal/anal and lung cancers showing broader variability (**Figure 7**). Correctness displayed the clearest differentiation: prostate and brain tumors achieved the highest median scores (≥3.5/4), breast and metastases performed intermediately, while rectal/anal and lung cancers scored lowest and most variably (**Figure 6**).

Beyond these main groupings, more granular subgroup analyses revealed clear data-defined differences. Highest correctness was observed in *prostate, intermediate risk* (correctness 3.83; comprehensiveness 3.83; hallucinations 0%) and *prostate, biochemical recurrence after RPE* (3.67; 3.83; 0%), as well as *small-cell lung cancer (SCLC)* (3.67; 4.00; 0%). Brain primaries were generally strong: *meningioma* (3.67; 3.67; 0%), *vestibular schwannoma* (3.67; 3.33; 0%), *glioma grade 2/3* (3.50; 3.67; 0%), and *glioblastoma* (3.33; 3.83; 0%); a weaker brain subgroup was *pituitary adenoma* (2.67; 3.50; 12.5%).

Breast adjuvant scenarios were solid for correctness and highly complete but showed higher hallucination rates in several subgroups: *adjuvant low risk* (3.33; 4.00; 25%), *adjuvant node-negative* (3.50; 4.00; 25%), *adjuvant node-positive* (3.17; 3.50; 12.5%), *DCIS* (3.00; 3.33; 12.5%), and *loco-regional recurrence* (3.00; 3.33; 0%).

Within lung cancer, *NSCLC stage III (definitive)* performed moderately (3.33; 3.44; 8.33%), whereas settings requiring finer adaptation were lower: *NSCLC re-irradiation* (2.54; 3.50; 12.5%) and *NSCLC SBRT* (2.78; 3.22; 25%).

For metastatic disease, *palliative bone metastases* performed well (3.67; 3.89; 0%) and *metastatic SBRT* was acceptable (3.11; 3.33; 0%), while *brain metastases* were lower and more error-prone (2.67; 3.33; 18.75%).

Rectum–anal cases were the weakest overall: *anal cancer* (3.00; 3.00; 0%), *neoadjuvant rectal cancer* (2.75; 3.25; 25%), and *local recurrence* (2.33; 3.56; 25%).

These patterns localize remaining challenges to problem settings that demand precise trial knowledge, dose/fractionation choices, or complex multimodality sequencing.

Several representative cases illustrate these limitations:

- Case 7 (low-risk prostate cancer): The system recommended definitive therapy, which some raters considered overtreatment given active surveillance would also have been guideline-concordant, though others accepted it because the patient requested therapy.
- Case 8 (neoadjuvant rectal cancer): Biomarker analysis (e.g. MSI) was omitted, a gap increasingly relevant for therapy planning.
- Case 11 (brain metastases): The combination of ipilimumab and nivolumab was proposed, but with a non–guideline-concordant dosing scheme, lowering correctness.
- Case 17 (DCIS): A radiotherapy boost dose was recommended, which is not guideline-supported and was judged overtreatment.
- Case 29 (NSCLC with SBRT): Systemic therapy options were suggested despite definitive SBRT being the standard in this context.
- Case 36 (lung SBRT): Chemotherapy cycles were not specified, reducing precision despite an otherwise acceptable plan.
- Case 42 (brain metastases): Therapy recommendations were given without histologic confirmation, a prerequisite step that reduced guideline adherence.
- Case 58 (SBRT): Outdated regimens were mixed with correct ones, producing polarized ratings.

To further clarify the nature of GPT-5 errors, we systematically categorized them by severity. At the lowest ranking level (mean score = 2, n = 5), major errors occurred, including three cases with incorrect radiotherapy recommendations and two cases with incorrect systemic therapy suggestions. At the intermediate ranking level (scores between 2 and 3, n = 14), errors were more nuanced: five cases included only one of several valid therapeutic options without clear justification, one case recommended a radiotherapy boost that is not guideline-concordant, two cases suggested non-standard chemotherapy regimens, one case contained a radiotherapy dosage error, and five systemic therapy recommendations contained minor inaccuracies. At the higher ranking level (scores ≥ 3, n = 41), correctness was generally preserved, but inconsistent issues remained, such as non-standard dosing of bisphosphonate therapy, omission of important management strategies (e.g., watch-and-wait approaches), and lack of clarification requests regarding essential patient information (e.g., performance status). This structured error categorization highlights that GPT-5’s shortcomings ranged from major treatment misassignments to more subtle omissions or deviations from guideline-concordant practice.

Where historical records permitted, GPT-5’s recommendations showed high concordance with delivered care across treatment intent, modality/technique, and dose/target ranges, although multiple reasonable options often existed.

## 4 Discussion

Large language models are poised to function as supervised amplifiers of clinical reasoning in radiation oncology, with their primary value lying not in test accuracy but in producing auditable, guideline-aware drafts that speed multidisciplinary deliberation while preserving human accountability. Real-world integration should prioritize governance (traceable evidence links and versioning), tool-coupled reasoning (retrieval and dose–constraint checks), and human-in-the-loop verification, particularly given persistent limits in visual reasoning. Our evaluation framework—combining standardized knowledge testing with expert-rated, real-world vignettes—offers a practical template for assessing clinical utility through explainability and error typologies rather than accuracy alone.

This work extends the literature on large language models (LLMs) in radiation oncology along two complementary axes: standardized examination performance and real-world decision support. On the ACR TXIT subset, the present model attained 92.8% under the same item pool and adjudication rules previously used for GPT-3.5/4, which yielded 63.1% and 74.1%, respectively (39). While this marks a substantial advance, such accuracy cannot be equated with clinical safety, as even a small residual error rate may translate into unacceptable risks without human oversight. The magnitude of the observed gain is consistent with the architectural and training changes that distinguish GPT-5 from its predecessors: scaling of transformer capacity, more stable long-context attention, preference optimization with richer feedback, and—critically—its explicit positioning as a reasoning model. Unlike GPT-3.5 and GPT-4, GPT-5 is designed not only to recall information but to generate structured, stepwise rationales, yielding more consistent and interpretable outputs (37, 38). Our use of a constrained response format (“Final answer: X”) reduced adjudication noise without contributing domain knowledge. Examination accuracy nevertheless remains an imperfect surrogate for clinical competence: persistent weaknesses were observed in brachytherapy, fine-grained dosimetry, and evolving trial-specific details, mirroring deficits documented for earlier models (39). In addition, GPT-5 correctly answered only 2 of 7 image-based TXIT items, underscoring its limited visual reasoning capabilities and highlighting that, despite progress in text-based reasoning, current models remain unreliable for image interpretation in radiation oncology.

Persistent weaknesses were noted in brachytherapy and trial-specific knowledge, likely reflecting limited representation of these domains in publicly available training data. Brachytherapy guidance is largely confined to specialized society documents, while trial protocols are often behind paywalls or scattered across conference proceedings, reducing their visibility to large-scale pretraining. Addressing these gaps will require domain-targeted fine-tuning and integration of curated trial databases or retrieval pipelines.

Comparisons with adjacent evaluations clarify where improvements reflect reasoning advances rather than test artifacts. In radiation oncology physics, Holmes et al. showed that item structure and distractors meaningfully influence performance (29), while Wang et al. demonstrated that simply shuffling answer options alters accuracy (30). Our reproduction of prior TXIT baselines with identical stems, options, and scoring therefore supports the interpretation that GPT-5’s higher scores reflect genuine advances in reasoning and knowledge synthesis rather than format effects. At the same time, gynecologic oncology, brachytherapy, and trial-anchored items remained more challenging, consistent with topic-level variability in prior reports (15, 16, 39).

A more robust and clinically relevant benchmark was established through our 60-case evaluation based on authentic oncologic vignettes, in which GPT-5 was tasked with generating structured management plans. The model produced coherent and comprehensive drafts, extending earlier findings with GPT-4 that had been reported in a smaller Red Journal–style Gray Zone cases (39). Hallucinations were infrequent and did not pose a substantive concern; rather, errors were concentrated in areas requiring detailed trial-specific knowledge, nuanced clinical adaptation or complex multimodality treatments (e.g., SBRT, DCIS, brain metastases, ano-rectal cancer, lung cancer with comorbidities). Inter-rater variability, while contributing to lower *κ* values (leiss’ *κ* = 0.083 for correctness, *κ* =−0.016 for comprehensiveness, and *κ* =−0.111 for hallucinations), similarly reflects the legitimate diversity of acceptable clinical strategies in these complex scenarios, underscoring that LLM outputs may reasonably diverge from one clinician’s judgment while still remaining guideline-concordant. Taken together, these results highlight the potential of reasoning-oriented models: GPT-5 is able to synthesize case-relevant rationales, thereby moving closer to providing the deliberative support that is directly applicable in tumor board settings.

A closer examination of hallucinations revealed that most were not “classic” fabrications, such as nonexistent drugs or spurious references, but rather subtler drifts within otherwise plausible recommendations. Hallucinations were reported by only one of the four raters and were usually related to apparent overtherapy without explicit justification. A notable example was the repeated suggestion of zoledronic acid 4mg IV every 6 months for 3 years. While this regimen exists in osteoporosis practice, it is not a recognized oncology schedule, where dosing intervals are typically every 3–12 weeks and duration is generally limited to 2 years with reassessment. Three of the four experts therefore judged these outputs as “incorrect” rather than as hallucinations, whereas one reviewer flagged them as such. This highlights that hallucinations in clinical LLM evaluation may span a spectrum—from minor factual drifts (plausible but guideline-incongruent regimens) to potentially harmful errors (overtreatment, misdosing, or omission of critical steps). Such misalignments could likely have been mitigated by more restrictive prompting (e.g., “use only oncology guidelines, not osteoporosis or other indications”), but this was deliberately omitted to avoid overfitting and to better approximate real-world deployment conditions.

Our evaluation complements emerging work such as the *Articulate Medical Intelligence Explorer (AMIE)* system, which was tested in synthetic breast oncology vignettes (33). While AMIE incorporated retrieval and self-critique pipelines and demonstrated performance above trainees and fellows, our study extends this line of research by benchmarking GPT-5 on real, anonymized, multi-disease radiation oncology cases rated by board-certified specialists.

Our findings align with broader medical LLM studies, which consistently show topic-level heterogeneity, stronger outputs under expert scaffolding, and improved interpretability when reasoning steps are made explicit (15, 16, 31, 32). Reviews and meta-analyses converge on supervised applications—education, tumor-board summarization, pre-board preparation—rather than autonomous decision-making (17, 18, 44). Within this trajectory, GPT-5’s positioning as a reasoning model represents a qualitative step: it enables explicit rationale generation and structured synthesis across complex oncology cases, something prior models handled only inconsistently.

Methodologically, our study also connects to emerging multimodal planning assistants that couple LLM reasoning to imaging or dose engines (27, 28). Such systems may compensate for GPT-5’s current blind spots in image review and dosimetry but will require rigorous validation and regulatory oversight before clinical use (15, 45). From a governance perspective, near-term deployment should remain supervised, with retrieval-augmented pipelines, auditable links to guidelines and trials (e.g., AJCC, PORTEC-3, ORIOLE), explicit uncertainty labeling, and human sign-off (41–43, 45, 46).

Retrieval-augmented systems such as Med-PaLM 2 and AMIE combine generative reasoning with evidence retrieval and self-critique, achieving strong performance but relying on curated sources at inference time (33, 34). In contrast, GPT-5 in our study operated without retrieval and nevertheless showed high TXIT accuracy and solid vignette performance, suggesting it may represent a new baseline for model-internal reasoning. However, definitive conclusions require cross-platform comparisons on larger and more diverse datasets.

Beyond technical performance, deployment of GPT-5 raises ethical and regulatory considerations. If applied to diagnosis or treatment planning, the system could qualify as a medical device under EU MDR or FDA frameworks, yet it is not currently validated for such use. Until formal regulatory pathways are established, GPT-5 outputs should be regarded as educational or research aids, with responsible use requiring human oversight, auditable evidence links, and clear boundaries of autonomy.

An additional consideration is model versioning and potential drift across updates. Although we reported the exact model identifier and used standardized prompts with repeated runs, future versions may behave differently, complicating reproducibility of benchmark results. This underscores the need for transparent version reporting, archiving of raw outputs, and community benchmarks to monitor stability over time.

Future research should prioritize prospective studies, ideally randomizing tumor-board workflows to model-assisted versus control arms. Additional directions include systematic evaluation of reasoning under tool use and retrieval conditions, direct comparisons of general-purpose and domain-adapted models, explicit assessment of target and dose coherence as well as toxicity forecasting, and development of auditable retrieval pipelines tightly coupled to primary evidence (18). Ultimately, the goal is not to replace clinician judgment, but to generate reproducible, evidence-linked drafts and option sets that accelerate multidisciplinary deliberation while preserving safety, accountability, and trust.

## 5 Limitations

Several limitations frame the interpretation of our findings. First, standardized examinations sample knowledge differently from real-world practice; thus, high TXIT performance does not guarantee reliability in rare, ambiguous, or evolving scenarios (13). Second, the retrospective vignette cohort—while enriched by follow-up and therapy verification—may be affected by survivorship and documentation biases. Third, despite prespecified rubrics, inter-rater variability persisted, reflecting the complexity and plurality of oncologic decision-making (17). Fourth, GPT-5 was tested in isolation, without external tools; integration with guideline retrieval, dose–constraint engines, toxicity predictors, or trial databases could plausibly improve accuracy, safety, and auditability. Fifth, the TXIT and 60-case sets cover only a subset of radiation oncology knowledge, and cannot capture the full diversity of decision-making. Finally, model versioning, decoding parameters, and memory constraints may influence outputs; these were mitigated through fresh sessions and repeated runs, but residual variability remains.

## 6 Conclusion

On TXIT benchmarks, GPT-4 outperformed GPT-3.5, and GPT-5 further increased accuracy to 92.8%, with strengths in statistics, CNS/eye, physics, diagnostic methods, and toxicity, and persistent gaps in gynecology, brachytherapy, dosimetry, and trial-specific details.

More importantly, our novel 60-case benchmark of real-world oncologic scenarios showed that GPT-5 can generate coherent, comprehensive management drafts. Hallucinations were rare and not a substantive concern; limitations instead reflected occasional inaccuracies in guideline details, multimodal treatments, and nuanced adaptation of medical knowledge to complex clinical case descriptions.

In the near term, GPT-5 is best positioned for supervised applications: education and pre-board preparation; structured draft generation for tumor boards (with traceable evidence links and human sign off); and standardized documentation support. These uses exploit its strength in fast, coherent synthesis while keeping clinicians in control of final decisions.

Future applications may include semi-autonomous decision support and tighter integration with external tools (retrieval of up-to-date guidelines and trials, dose–constraint engines, toxicity predictors). Realizing this will require prospective evaluation, robust safety guardrails (uncertainty disclosure, override pathways), auditability, and—where applicable—regulatory clearance. Until such conditions are met, GPT-5 should be deployed as an augmentative assistant rather than an autonomous decision-maker.

## Data Availability

The raw data supporting the conclusions of this article will be made available by the authors, without undue reservation.

## Supplementary material

Supplementary Table 1 Full list of benchmark cases included in the real-world oncologic decision-support evaluation.
