# Correction: Benchmarking GPT-5 in radiation oncology: measurable gains, but persistent need for expert oversight

**DOI:** 10.3389/fonc.2025.1766619

**Published:** 2026-01-06

**Authors:** Ugur Dinç, Jibak Sarkar, Philipp Schubert, Sabine Semrau, Thomas Weissmann, Andre Karius, Johann Brand, Bernd-Niklas Axer, Ahmed Gomaa, Pluvio Stephan, Ishita Sheth, Sogand Beirami, Annette Schwarz, Udo Gaipl, Benjamin Frey, Christoph Bert, Stefanie Corradini, Rainer Fietkau, Florian Putz

**Affiliations:** 1 Department of Radiation Oncology, University Hospital Erlangen, Friedrich-Alexander-Universität Erlangen-Nürnberg, Erlangen, Germany; 2 Comprehensive Cancer Center Erlangen-Europäische Metropolregion Nürnberg (European Metropolitan Region Nuremberg) (CCC ER-EMN), Erlangen, Germany; 3 Bavarian Cancer Research Center (BZKF), Munich, Germany; 4 Department of Radiation Oncology, University Hospital, Ludwig Maximilian University of Munich, Munich, Germany

**Keywords:** GPt-5, artificial intelligence, radiation oncology, large language models, treatment recommendation, oncologic decision support, hallucination, real-world evaluation

Author “Ugur Dinç” was erroneously spelled as “Udo S. Dinc”.

The original version of this article has been updated.

