# Supplementary material for: Benchmarking GPT-5 in radiation oncology: measurable gains, but persistent need for expert oversight
Source: Front Oncol. 2025 Dec 11;15:1695468. doi: 10.3389/fonc.2025.1695468 (PMC12738326; doi:10.3389/fonc.2025.1695468)
Supplement: Supplementary Table 1 — Full list of benchmark cases included in the real-world oncologic decision-support evaluation. [file Supplementaryfile1.pdf]

Supplementary Material

1 SUPPLEMENTARY DATA

Listing 1. The exact prompt/function used for clinical vignettes:

```
 f"""
You are a tumor board assistant in Germany (radiation oncology, medical
   oncology, surgical, ENT).
Cite German S3 guidelines first with exact recommendation numbers and short
   direct quotes.
Use secondary sources (NCCN/ESMO/ESTRO/ICRU) only as supportive.

Output ONE SINGLE LINE of JSON with EXACTLY these keys:

- "diagnosis_compact": three short lines, each very concise:
    Line 1: cancer type + side/site (if known) + ED:MM/YY (Erstdiagnose),
   and if applicable: Rez MM/YY (Rezidivmonat/-jahr)
        e.g., "Mammakarzinom links ED:05/2023, Rez 03/2025"
    Line 2: TNM (c/p + T,N,M), key biology (e.g., ER/PR, HER2, p16/HPV, RAS/
   BRAF, MSI/MSS, Gx)
        e.g., "cT2 cN1 cM0, ER/PR+, HER2, G2"
    Line 3: starts with "Bisherige onkologische Therapie:" + last relevant
   procedure/systemic therapy, very compact
        e.g., "Bisherige onkologische Therapie: Zn. OP (BET) 06/2025" or "
   Bisherige onkologische Therapie: Zn. 6x Cis/Pembro"
- "therapy_compact": one concise line (German abbreviations) like:
    "adj. RCT: 50 Gy/25 Fr (ED 2.0 Gy) + Boost 10 Gy/5 Fr; Chemo: CAPOX q21d
    3"
- "tumorformel": concise TNM/tumor formula if inferable; otherwise "Unklar".
- "suggested_therapieplan": concrete, guideline-aligned plan. If listing
   multiple points,
  put each point on its own line starting with a number and a closing
   parenthesis,
  e.g., "1) First thing\\n2) Second thing\\n3) Third thing".
  Cover: chemotherapy (drug(s), schema, dose or range), radiotherapy (
   technique, target volumes, total dose & fractionation),
  surgery (if/when appropriate), tumorboard recommendation.
- "notes_to_clinician": practical next steps (further diagnostics before/
   after/during, labs, imaging, pathology, p16/HPV if HNSCC,
  renal/hepatic clearance, dental eval, PEG, toxicity considerations). Use
   numbered new lines as above.
- "guideline_primary": German S3 priority list with each item on its own
   line, format:
  "1) S3 [Disease], Empfehlung Nr. X.Y: \\"short quote\\""
- "guideline_secondary": secondary brief support with each item on its own
   line, format:
  "1) NCCN v.2025.1: short point" or "2) ESTRO/ICRU: short point"
- "key_characteristics": numbered list (each on its own line) stating:
  [Therapieelement]  Indikation: [kurze Begrndung basierend auf
   Patientendaten/Guidelines]
  e.g., "1) RT 70 Gy  Indikation: definitive Behandlung bei lokal
   fortgeschrittenem HNSCC (cT3N2bM0)"
- "self_score": integer 0100 reflecting confidence in the suggestions given
   the available data
```

```
  (100 = guideline-clear with complete info; lower if key data are missing/
   ambiguous). Return only the number.

Rules:
- Output JSON ONLY (no prose before/after).
- If critical info is missing, say what's needed in "notes_to_clinician" and
    give a conservative provisional plan.

Patient INPUT (verbatim):
randID: {rand_id}
Age: {age}
Diagnose: {diagnose}
Nebendiagnosen: {nebendiagnosen}
Anamnese: {anamnese}
""".strip()
```

Listing 2. The exact prompt/function used for multiple choice questions: :

```
def build_user_content(question_text: str, image_path: str | None):
    parts = [
        {"type": "input_text", "text": (
            "Please answer the following ACR multiple-choice exam question.
   "
            "First answer comprehensively deriving from your expert
   knowledge, "
            'then give the final answer in the following form: "Final answer
   : X" '
            "where X is A, B, C, or D.\n\n"
            f"ACR question:\n{question_text}\n"
        )}
    ]
    if image_path:
        with open(image_path, "rb") as f:
            b64 = base64.b64encode(f.read()).decode("ascii")
        parts.append({
            "type": "input_image",
            "image_url": f"data:image/png;base64,{b64}",
        })
    return parts
```

2 SUPPLEMENTARY TABLES AND FIGURES

Supplementary Table S1: Full list of benchmark cases included in the real-world oncologic decision-support evaluation.

Case #	Tumor Site	Clinical Scenario
1	Rectum-Anal	Neoadjuvant – Rectal Cancer
2	Rectum-Anal	Neoadjuvant – Rectal Cancer
3	Lung	NSCLC – SBRT
4	Breast	Adjuvant – nodal positive
5	Brain	Meningioma
6	Breast	Adjuvant – nodal positive
7	Prostate	Definitive – low risk
8	Rectum-Anal	Neoadjuvant – Rectal Cancer
9	Lung	SCLC
10	Rectum-Anal	Neoadjuvant – Rectal Cancer
11	Metastases	Brain metastases
12	Lung	NSCLC – Definitive – Stage III
13	Breast	DCIS
14	Prostate	Definitive – high risk
15	Brain	Glioma grade 2 / 3
16	Brain	Vestibular Schwannoma
17	Breast	DCIS
18	Brain	Pituitary Adenoma
19	Lung	NSCLC – Definitive – Stage III
20	Rectum-Anal	Anal Cancer
21	Breast	Loco-regional recurrence
22	Brain	Glioblastoma grade 4
23	Rectum-Anal	Local Recurrence
24	Brain	Vestibular Schwannoma
25	Prostate	Definitive – high risk
26	Lung	NSCLC – Re-RT
27	Prostate	Local recurrence after RPE + EBRT
28	Rectum-Anal	Local Recurrence
29	Lung	NSCLC – SBRT
30	Prostate	Local recurrence after RPE + EBRT
31	Brain	Glioblastoma grade 4
32	Prostate	Definitive – low risk
33	Lung	NSCLC – SBRT
34	Prostate	Definitive – intermediate risk
35	Brain	Pituitary Adenoma
36	Metastases	SBRT
37	Lung	NSCLC – Re-RT
38	Metastases	Brain metastases
39	Metastases	SBRT
40	Lung	NSCLC – Definitive – Stage III
41	Prostate	Biochemical recurrence after RPE
42	Metastases	Brain metastases
43	Breast	Adjuvant – low-risk
44	Lung	SCLC
45	Metastases	Palliative – bone metastases
46	Prostate	Biochemical recurrence after RPE
47	Rectum-Anal	Local Recurrence
48	Metastases	Palliative – bone metastases
49	Metastases	Palliative – bone metastases

Case #	Tumor Site	Clinical Scenario
50	Metastases	Brain metastases
51	Rectum-Anal	Anal Cancer
52	Prostate	Definitive – intermediate risk
53	Breast	Adjuvant – nodal negative
54	Breast	Adjuvant – nodal negative
55	Rectum-Anal	Anal Cancer
56	Breast	Adjuvant – low-risk
57	Brain	Glioma grade 2 / 3
58	Metastases	SBRT
59	Brain	Meningioma
60	Breast	Loco-regional recurrence
